# Trends and age-related characteristics of substance use in the hospitalized homeless population

**DOI:** 10.1097/MD.0000000000028917

**Published:** 2022-02-25

**Authors:** Sung-youn Chun, Ji W. Yoo, Hyeki Park, Jinwook Hwang, Pearl C. Kim, Seong Park, Jay J. Shen

**Affiliations:** a Research and Analysis Team, National Health Insurance Service Ilsan Hospital, Goyang, Republic of Korea; b Department of International Cooperation, Health Insurance Review & Assessment Service, Wonju, Republic of Korea; c Department of Internal Medicine, University of Nevada School of Medicine, Las Vegas, NV; d Department of Thoracic and Cardiovascular Surgery, Korea University Ansan Hospital, Korea University College of Medicine, Seoul, Republic of Korea; e Department of Healthcare Administration and Policy, University of Nevada Las Vegas School of Public Health, Las Vegas, NV; f Department of Criminal Justice, University of Nevada Greenspun College of Urban Affairs, Las Vegas, NV.

**Keywords:** homeless, hospitalisation, substance use

## Abstract

We aimed to examine trends and characteristics of substance use (opioid, cocaine, marijuana, and heroin) among hospitalized homeless patients in comparison with other hospitalized patients in 3 states.

This was a cross-sectional study, based on the 2007 to 2015 State Inpatient Data of Arizona, Florida, and Washington (n = 32,162,939). Use of opioid, cocaine, marijuana, heroin, respectively, was identified by the International Classification of Diseases, 9th Revision. Multi-level multivariable regressions were performed to estimate relative risk (RR) and 95% confidence intervals (CI). Dependent variables were the use of substances (opioid, cocaine, marijuana, and heroin), respectively. The main independent variable was homeless status. The subgroup analysis by age group was also conducted.

Homeless patients were associated with more use of opioid (RR [CI]), 1.23 [1.20–1.26], cocaine 2.55 [2.50–2.60], marijuana 1.43 [1.40–1.46], and heroin 1.57 [1.29–1.91] compared to other hospitalized patients. All hospitalized patients including those who were homeless increased substance use except the use of cocaine (RR [CI]), 0.57 [0.55–0.58] for other patients and 0.60 [0.50–0.74] for homeless patients. In all age subgroups, homeless patients 60 years old or older were more likely to be hospitalized with all 4 types of substance use, especially, cocaine (RR [CI]), 6.33 [5.81–6.90] and heroin 5.86 [2.08–16.52] in comparison with other hospitalized patients.

Homeless status is associated with high risks of substance use among hospitalized patients. Homeless elderly are particularly vulnerable to use of hard drugs including cocaine and heroin during the opioid epidemics.

## 1 Introduction

In the United States (U.S.), approximately 1% of the population experiences homeless or housing instability every year with estimation of more than 600,000 homeless individuals every night.^[1]^ The number of homeless individuals has increased in the metropolitan areas as the growing mismatch of incomes and housing costs.^[2]^ The U.S. Interagency Council on Homelessness reported an increase in the number of homeless individuals, especially, the numbers of children and families, during the past 3 decades.

The association between socioeconomic status and health outcomes is well established.^[3,4]^ Homeless people are in a high risk of being overburdened by medical and mental illnesses and use acute health care at high rates.^[4]^ Homeless people often have relatively poorer health status than general population,^[5]^ often resulting from multiple risk factors, such as financially constrained access to appropriate health care and less hygienic conditions.^[6,7]^ Recent studies have described improper health behaviors as a major cause of poor health outcomes among homeless people. Furthermore, the high prevalence of substance use disorders in homeless populations is well recognized.^[3,8]^ Substance use remains primary drivers of hospitalization among the homeless population.^[9]^

The epidemic of opioid abuse and dependency over the past 2 decades has become a major health policy and public health concern in the U.S. Over 70,000 people died from drug overdose in 2017.^[10]^ The drug epidemic has manifested in many ways. For example, hepatitis C virus infections increased 3-folds from 2010 to 2016, primarily among young adults aged <40 years.^[11]^ Heroin-related deaths increased more than 5-folds over a 7-year period from 2010 to 2017.^[10]^ The epidemic is so far-reaching that it has been cited as a contributing factor to the declining life expectancy of the nation.^[12]^ While more diseases and deaths are occurring related to use of opioids more than ever, more states have eased regulations on medical and recreational marijuana use. As a result, the marijuana-related emergency department visits increased annually by 7% in the U.S.^[13]^

There are myriad ways in homelessness poses risk of substance use.^[4]^ Emergency shelters are often overcrowded, without enough bedding and bathroom facilities. Unsheltered environments lead homeless individuals with mental illness to a high risk of substance abuse and dependence unless underlying mental health conditions are appropriately treated.^[14]^ Homeless individuals with mental health conditions are more vulnerable to escalating risks of sexually transmitted diseases, for example, hepatitis B or C, as well as human immunodeficiency virus infection. According to the Boston cohort study of homeless individuals, they are 8 to 17 times more likely to experience drug overdose death, compared to the general population.^[15,16]^ Homeless individuals are observed to have “accelerated aging” process compared to the general population as homeless individuals have higher prevalence of geriatrics syndromes (impaired cognition and activities of daily living) compared to their counterparts.^[17]^ Over the past 2 decades, the number of homeless individuals aged 50 and older has quadrupled.^[18]^ Older homeless individuals are more susceptible to interactions between substance use and their medications for chronic medical conditions. According to a population-based survey for older homeless individuals, almost two thirds had dependency in at least 1 substance use.^[19]^ Nonetheless, the research has been limited to examine large scale trends and demographics of substance use among hospitalized homeless patients, during the opioid epidemics. Therefore, this study, using large multi-state datasets, aimed to examine the trends in substance-related hospitalizations of homeless individuals compared to other hospitalized patients and demographic characteristics especially the age factor of substance use among hospitalized homeless patients.

## 2 Methods

### 2.1 Data source and study population

The state inpatient database (SID), a publically available dataset, was used in this study. SID contains hospital discharge records of all community hospitals in the participating states, and it was originally developed for the Healthcare Cost and Utilization Project (HCUP) by Agency for Healthcare Research and Quality (AHRQ). SID of all participating states cover more than 95% of all U.S. hospital discharge. The SID includes an anonymous patient-level information including demographics, diagnostic codes and procedures.^[20]^ We used data from 3 states’ SID; they are Arizona, Florida, and Washington. This study included all hospital records in which homeless information was recorded among the 3 sates of SID from the first quarter of 2007 to the third quarter of 2015. We excluded Arizona 2007 and Washington from 2007 to 2009 due to missing the homeless variable. After removing other missing values, the final number in our analysis was 32,065,120. The study was approved by the Institution Review Board of University of Nevada at Las Vegas.

### 2.2 Dependent variable

There were 4 dichotomous dependent variables defined as use of a specific substance (ie, opioid, cocaine, marijuana, and heroin), respectively, with a value of “1” indicating Yes and “0” indicating No. Substance use was defined as abuse, dependence, unspecified use, based on the International Classification of Diseases, 9^th^ edition, clinical modification codes in the Table S1, Supplemental Digital Content in accordance to the HCUP data notes and methods (HCUP fast facts: opioid-related hospital use. Healthcare Cost and Utilization Project. October 2018. AHRQ, Rockville, MD. https://www.hcup-us/ahrq.gov/fastfacts/opioid/opioiduse.jsp.).

### 2.3 Independent variable

The independent variable was homeless status that was collected from the SID dataset. Homeless variable was constructed from data reported by hospitals, and this data does not distinguish whether they are chronically homeless or not and sheltered homeless or not.^[20]^

### 2.4 Covariates

They included demographic and health related variables. For demographic variables, we used sex (male or female), age (<20 years, 20–39 years, 40–59 years, 60+ years old), ethnicity/race (White, Black, Hispanic, Asian, and other), state of the hospital (Arizona, Florida, Washington), mental health conditions, and number of comorbidities (low 0–1, mid 2–3, high 4 and above). Mental health conditions were defined as mood disorders, schizophrenia, other nonmood psychotic disorders, anxiety, stress-related, somatoform disorders, personality and factitious disorders in the Supplemental Digital Content 1. The number of comorbidities was comprised by the AHRQ comorbidity measures provided by AHRQ in SID, and it identifies coexisting medical conditions that are not directly related to the principal diagnosis. It includes medical conditions as follow: (acquired immune deficiency syndrome, alcohol abuse, deficiency anemias, rheumatoid arthritis/collagen vascular diseases, chronic blood loss anemia, congestive heart failure, chronic pulmonary disease, coagulopathy, depression, diabetes without complication, diabetes with chronic complications, hypertension uncomplicated, hypertension complicated, hypothyroidism, liver disease, lymphoma, fluid and electrolyte disorders, metastatic cancer, other neurological disorders, obesity, paralysis, peripheral vascular disorders, psychoses, pulmonary circulation disorders, renal failure, solid tumor without metastasis, peptic ulcer disease excluding bleeding, valvular disease, and weight loss.^[20]^ Also, we included marijuana legalization (before, after) to adjust potential variations resulting from states’ legalization statuses.

### 2.5 Statistical analysis

The characteristics and drug-related hospitalization rates between homeless individuals and other hospitalized patients were examined using *t* test. The general estimating equation Poisson model with log link^[21]^ was used to examine the effect of homeless status on substance-related hospitalizations by substance type after adjusting the covariates. Estimation was calculated as relative risk (RR) and 95% confidence intervals (CIs). To investigate time trends of drug-related hospitalizations, multi-level multivariate regressions were performed. In addition, another subgroup analysis was performed based on different age groups (younger than 20 years, 20–39 years, 40–59 years, 60 years or older) to explore the age-related characteristics of substance-related hospitalizations. Analyses were adjusted for all covariates and *P* < .05 was considered statistically significant. Analysis was performed using the SAS software, version 9.4 (SAS Institute, Cary, NC).

## 3 Results

Characteristics of homeless patients and other patients are listed in the Supplemental Digital Content 2. Among 32,065,120 hospitalizations, homeless status was 0.30% (97,819 hospitalizations). Among the homeless individuals, most were males (78.34%). Mental health conditions accounted for 33.93%. Most of homeless hospitalizations were from state of Florida (90.85%). Table 1 presents the characteristics of substance use hospitalizations by substance types. In opioid use, homeless individuals were more frequently hospitalized compared to all other hospitalized patients (6.39% vs 1.57%). The same pattern was observed in the use of cocaine (12.54% vs 0.98%), marijuana (8.84% vs 1.30%), and heroin (0.11% vs 0.02%). The Supplemental Digital Contents 3 to 6 present the unadjusted annual rate of opioid, cocaine, marijuana, and heroin-related hospitalization per total 100,000 hospitalizations of homeless and general population. Substance use hospitalization rates monotonically increased every in all type of substance use. In opioid and marijuana-related hospitalization rate, the slope of the graph was steadily higher in homeless (Figures S1 and S3, Supplemental Digital Content). Rate of the cocaine abuse admission decreased at first but increased slightly in the end in both homeless and general population (Figure S2, Supplemental Digital Content). However, fluctuation was bigger in homeless. In heroin abuse admission rate, unstable graph was shown among homeless, but rate of the admission and slope was still higher in homeless individuals.

**Table 1:** Patient characteristics of use of substances by substance type.

	Opioid					Cocaine					Marijuana					Heroin				
	Yes		No		*P*	Yes		No		*P*	Yes		No		*P*	Yes		No		*P*
	N	%	N	%		N	%	N	%		N	%	N	%		N	%	N	%	
Homeless status																				
Nonhomeless	504,697	1.57	31,560,423	98.43	<.001	314,690	0.98	31,750,430	99.02	<.001	417,180	1.30	31,647,940	98.70	<.001	5581	0.02	32,059,539	99.98	<.001
Homeless	6247	6.39	91,572	93.61		12,269	12.54	85,550	87.46		8651	8.84	89,168	91.16		104	0.11	97,715	99.89	
Time (yr)																				
2007	21,317	0.84	2,520,853	99.16	<.001	34,588	1.36	2,507,582	98.64	<.001	22,294	0.88	2,519,876	99.12	<.001	172	0.01	2,541,998	99.99	<.001
2008	32,733	0.98	3,291,738	99.02		36,253	1.09	3,288,218	98.91		30,073	0.90	3,294,398	99.10		266	0.01	3,324,205	99.99	
2009	38,577	1.15	3,326,905	98.85		32,558	0.97	3,332,924	99.03		33,435	0.99	3,332,047	99.01		282	0.01	3,365,200	99.99	
2010	56,450	1.44	3,872,674	98.56		34,239	0.87	3,894,885	99.13		41,650	1.06	3,887,474	98.94		428	0.01	3,928,696	99.99	
2011	67,925	1.71	3,908,062	98.29		37,422	0.94	3,938,565	99.06		48,046	1.21	3,927,941	98.79		509	0.01	3,975,478	99.99	
2012	70,722	1.78	3,904,516	98.22		37,496	0.94	3,937,742	99.06		54,011	1.36	3,921,227	98.64		667	0.02	3,974,571	99.98	
2013	74,260	1.88	3,875,085	98.12		39,547	1.00	3,909,798	99.00		61,149	1.55	3,888,196	98.45		866	0.02	3,948,479	99.98	
2014	81,077	2.01	3,948,264	97.99		41,094	1.02	3,988,247	98.98		71,487	1.77	3,957,854	98.23		1163	0.03	4,028,178	99.97	
2015	67,883	2.21	3,003,898	97.79		33,762	1.10	3,038,019	98.90		63,686	2.07	3,008,095	97.93		1332	0.04	3,070,449	99.96	
Sex																				
Male	256,260	1.83	13,775,145	98.17	<.001	209,266	1.49	13,822,139	98.51	<.001	268,308	1.91	13,763,097	98.09	<.001	3960	0.03	14,027,445	99.97	<.001
Female	254,684	1.40	17,876,850	98.60		117,693	0.65	18,013,841	99.35		157,523	0.87	17,974,011	99.13		1725	0.01	18,129,809	99.99	
Age																				
<20 yr-old	16,692	0.34	4,944,568	99.66	<.001	7284	0.15	4,953,976	99.85	<.001	56,744	1.14	4,904,516	98.86	<.001	276	0.01	4,960,984	99.99	<.001
20–39 yr-old	211,399	3.55	5,747,645	96.45		120,141	2.02	5,838,903	97.98		207,273	3.48	5,751,771	96.52		3670	0.06	5,955,374	99.94	
40–59 yr-old	205,344	2.91	6,854,313	97.09		179,568	2.54	6,880,089	97.46		138,863	1.97	6,920,794	98.03		1550	0.02	7,058,107	99.98	
60 + yr-old	77,509	0.55	14,105,469	99.45		19,966	0.14	14,163,012	99.86		22,951	0.16	14,160,027	99.84		189	0.001	14,182,789	100.00	
Ethnicity/race																				
White	419,436	1.96	20,951,747	98.04	<.001	159,438	0.75	21,211,745	99.25	<.001	257,841	1.21	21,113,342	98.79	<.001	4450	0.02	21,366,733	99.98	<.001
Black	33,841	0.79	4,237,336	99.21		117,325	2.75	4,153,852	97.25		99,250	2.32	4,171,927	97.68		227	0.005	4,270,950	99.99	
Hispanic	42,569	0.83	5,062,379	99.17		41,107	0.81	5,063,841	99.19		51,181	1.00	5,053,767	99.00		784	0.02	5,104,164	99.98	
Asian and other	15,098	1.07	1,400,533	98.93		9089	0.64	1,406,542	99.36		17,559	1.24	1,398,072	98.76		224	0.02	1,415,407	99.98	
Mental health conditions																				
No	289,761	1.03	27,880,040	98.97	<.001	203,526	0.72	27,966,275	99.28	<.0001	257,862	0.92	27,911,939	99.08	<.001	4060	0.01	28,165,741	99.99	<.001
Yes	221,183	5.54	3,771,955	94.46		123,433	3.09	3,869,705	96.91		167,969	4.21	3,825,169	95.79		1625	0.04	3,991,513	99.96	
Number of co-morbidities																				
High (4 or more)	162,135	2.18	7,276,329	97.82	<.0001	103,832	1.40	7,334,632	98.60	<.001	94,451	1.27	7,344,013	98.73	<.001	1451	0.02	7,437,013	99.98	<.001
Medium (2 or 3)	197,889	2.00	9,707,728	98.00		153,323	1.55	9,752,294	98.45		200,766	2.03	9,704,851	97.97		2906	0.03	9,902,711	99.97	
Low (0 or 1)	150,920	1.02	14,667,938	98.98		69,804	0.47	14,749,054	99.53		130,614	0.88	14,688,244	99.12		1328	0.01	14,817,530	99.99	
Marijuana legalization																				
After	260,460	2.10	12,130,770	98.73	<.001	104,852	0.85	12,286,378	99.15	<.001	208,366	1.68	12,182,864	98.32	<.001	4377	0.04	12,386,853	99.96	<.001
Before	250,484	1.27	19,521,225	97.90		222,107	1.12	19,549,602	98.88		217,465	1.10	19,554,244	98.90		1308	0.01	19,770,401	99.99	
State																				
Arizona	105,546	1.78	5,810,607	98.22	<.001	34,023	0.58	5,882,130	99.42	<.001	83,917	1.42	5,832,236	98.58	<.001	1660	0.03	5,914,493	99.97	<.001
Florida	316,515	1.38	22,702,684	98.62		275,448	1.20	22,743,751	98.80		290,487	1.26	22,728,712	98.74		2382	0.01	23,016,817	99.99	
Washington	88,883	2.75	3,138,704	97.25		17,488	0.54	3,210,099	99.46		51,427	1.59	3,176,160	98.41		1643	0.05	3,225,944	99.95	
**Total**	510,944	1.59	31,651,995	98.41		326,959	1.02	31,835,980	98.98		425,831	1.32	31,737,108	98.68		5685	0.02	32,157,254	99.98	

Results of the multivariable analysis are shown in Table 2. We observed the same direction of association across substance type, higher risks of substance use hospitalizations among homeless status (RRs and CIs were 1.23 [1.20–1.26] for opioid; 2.55 [2.50–2.60] for cocaine; 1.43 [1.40–1.46] for marijuana; 1.57 [1.29–1.91] for heroin) compared to the other patients counterparts. In opioid and marijuana, a trend of monotonic increases was shown (RRs and CIs were 2.05 [2.01–2.09] for opioid; 1.97 [1.94–2.01] for marijuana) in 2015 compared to the reference year in 2007. In cocaine, a trend of steady decline was shown (RRs and CIs, 0.57 [0.55–0.58]) in 2015 compared to the reference year in 2007. In heroin, there was no consistent directionality of time trends. Male, age 20 to 39 years-old, mental health conditions, high number of comorbidities, and Washington state patients tended to be consistently associated with the use of all type substance except for cocaine use in Florida. White patients were more likely to use opioid and heroin. Minorities were more likely associated with the use of cocaine compared to White patients.

**Table 2:** Factors associated with use of substance by substance type in hospitalizations.^∗.^

	Opioid			Cocaine			Marijuana			Heroin		
	RR	95% CI		RR	95% CI		RR	95% CI		RR	95% CI	
		Low	High		Low	High		Low	High		Low	High
Homeless (reference = nonhomeless)	1.23	1.20	1.26	2.55	2.50	2.60	1.43	1.40	1.46	1.57	1.29	1.91
Year (reference = 2007)												
2008	1.09	1.07	1.11	0.89	0.87	0.90	1.02	1.00	1.03	1.05	0.86	1.27
2009	1.24	1.22	1.26	0.73	0.72	0.74	1.10	1.08	1.12	1.11	0.91	1.34
2010	1.45	1.43	1.48	0.66	0.65	0.67	1.16	1.14	1.18	0.60	0.50	0.73
2011	1.65	1.63	1.68	0.68	0.67	0.69	1.26	1.24	1.28	0.67	0.56	0.81
2012	1.69	1.66	1.71	0.65	0.64	0.66	1.39	1.37	1.41	0.88	0.73	1.05
2013	1.76	1.73	1.78	0.66	0.65	0.67	1.55	1.53	1.58	1.15	0.96	1.38
2014	1.93	1.89	1.96	0.57	0.56	0.58	1.76	1.73	1.80	0.99	0.82	1.20
2015	2.05	2.01	2.09	0.57	0.55	0.58	1.97	1.94	2.01	1.44	1.19	1.74
Sex (reference = female)												
Male	1.41	1.40	1.41	2.04	2.03	2.06	2.30	2.28	2.31	3.48	3.29	3.69
Age (reference = less than 20)												
20–39	6.28	6.18	6.38	7.11	6.94	7.29	1.56	1.55	1.58	6.22	5.47	7.07
40–59	3.27	3.22	3.33	6.71	6.55	6.87	0.53	0.52	0.54	1.11	0.96	1.27
60 or more	0.65	0.63	0.66	0.53	0.52	0.55	0.06	0.06	0.06	0.06	0.05	0.08
Ethnicity/race (reference = White)												
Black	0.33	0.33	0.34	2.78	2.75	2.80	1.37	1.36	1.38	0.18	0.16	0.20
Hispanic	0.45	0.44	0.45	1.29	1.28	1.31	0.74	0.73	0.75	0.62	0.58	0.67
Asian and other	0.53	0.52	0.54	1.06	1.04	1.09	0.90	0.88	0.91	0.48	0.42	0.55
Mental health conditions (reference = no)												
Yes	2.87	2.85	2.89	2.13	2.12	2.15	2.48	2.46	2.49	1.09	1.02	1.16
Number of co-morbidities (reference = Low (0, 1))												
High (4 or more)	2.77	2.75	2.79	3.36	3.33	3.40	3.02	2.99	3.05	5.27	4.86	5.73
Medium (2∼3)	1.92	1.91	1.94	2.71	2.68	2.73	3.16	3.14	3.19	4.61	4.30	4.95
Marijuana legalization (reference = before)												
After	0.91	0.90	0.92	1.13	1.11	1.15	0.98	0.96	0.99	3.05	2.71	3.43
State (reference = Washington)												
Arizona	0.75	0.75	0.76	1.03	1.01	1.05	0.95	0.94	0.96	0.64	0.60	0.69
Florida	0.60	0.59	0.61	2.00	1.96	2.04	0.79	0.78	0.80	0.41	0.38	0.44

CI = confidence intervals, RR = relative ratio. ^∗^All covariates were adjusted.

Table 3 presents the time trends of substance use by homeless status. We found important trends in odds of hospitalizations associated with uses of different substances. On average, opioid use-related hospitalizations increased twice from 2008 to 2015 in both homeless patients (2.38 [1.98–2.85]) and all other patients (2.05 [2.01–2.08]). Cocaine use-related hospitalizations decreased by approximately 40% in both homeless patients (0.60 [0.50–0.74]) and other patients (0.57 [0.55–0.58]). In marijuana, homeless status (2.49 [2.11–2.94]) was associated with sharper increase in hospitalizations compared to all other patients (1.97 [1.93–2.01]). In heroin, the trend pattern was fluctuated in both general population and homeless status.

**Table 3:** Time trends of hospitalizations in use of substances by substance type and homeless status.^∗.^

	Opioid			Cocaine			Marijuana			Heroin		
	RR	95% CI		RR	95% CI		RR	95% CI		RR	95% CI	
		Low	High		Low	High		Low	High		Low	High
	**Nonhomeless**											
Time (yr, reference = 2007)												
2008	1.09	1.07	1.11	0.89	0.87	0.90	1.01	0.99	1.03	1.06	0.87	1.29
2009	1.24	1.22	1.26	0.73	0.72	0.75	1.10	1.08	1.12	1.12	0.93	1.37
2010	1.45	1.43	1.48	0.66	0.65	0.67	1.16	1.14	1.18	0.61	0.50	0.74
2011	1.65	1.62	1.68	0.68	0.67	0.69	1.25	1.23	1.27	0.68	0.57	0.83
2012	1.69	1.66	1.71	0.65	0.64	0.66	1.39	1.37	1.41	0.89	0.74	1.08
2013	1.75	1.73	1.78	0.66	0.65	0.67	1.55	1.52	1.57	1.17	0.97	1.40
2014	1.92	1.89	1.96	0.57	0.56	0.58	1.76	1.72	1.79	1.01	0.84	1.23
2015	2.05	2.01	2.08	0.57	0.55	0.58	1.97	1.93	2.01	1.47	1.21	1.78

	Homeless											
Time (yr, reference = 2007)												
2008	1.21	1.03	1.42	0.84	0.78	0.91	1.24	1.10	1.41	0.72	0.23	2.26
2009	1.49	1.28	1.74	0.68	0.62	0.74	1.15	1.02	1.31	0.71	0.22	2.23
2010	1.63	1.41	1.89	0.68	0.63	0.74	1.23	1.09	1.39	0.75	0.26	2.15
2011	1.84	1.59	2.12	0.70	0.65	0.76	1.34	1.19	1.51	0.29	0.08	1.03
2012	1.70	1.48	1.96	0.68	0.63	0.73	1.58	1.41	1.77	0.37	0.12	1.18
2013	1.78	1.55	2.04	0.65	0.60	0.70	1.72	1.54	1.91	0.79	0.30	2.06
2014	2.20	1.83	2.63	0.59	0.48	0.71	2.27	1.92	2.67	0.28	0.08	1.00
2015	2.38	1.98	2.85	0.60	0.50	0.74	2.49	2.11	2.94	0.50	0.14	1.73

CI = confidence intervals, RR = relative ratio. ^∗^All covariates were adjusted.

Table 4 presents age subgroup analysis results of substance use hospitalizations by homeless status. In all age subgroups, homeless status was more likely associated with opioid, cocaine, and marijuana-related hospitalizations than the nonhomeless status. The use of cocaine and heroin among homeless patients were the most common among the age group 60 and older (6.33 [5.81–6.90] for cocaine; 5.86 [2.08–16.52] for heroin) compared to all other patients. The use of opioid and marijuana among homeless patients were consistently more common by 8% to 98% and 34% to 160% ranges, respectively, compared to all other patients.

**Table 4:** Associations between homeless status and use of substances in hospitalizations by substance type and age group.^∗.^

	Opioid			Cocaine			Marijuana			Heroin		
Age groups	RR	95% CI		RR	95% CI		RR	95% CI		RR	95% CI	
		Low	High		Low	High		Low	High		Low	High
<20 yr-old	1.98	1.57	2.49	2.85	2.27	3.59	1.71	1.52	1.93	2.02	0.28	14.53
20–39 yr-old	1.35	1.30	1.40	2.37	2.29	2.44	1.34	1.30	1.38	1.61	1.28	2.04
40–59 yr-old	1.08	1.04	1.12	2.62	2.56	2.68	1.54	1.49	1.59	1.33	0.89	1.99
60+ yr-old	1.95	1.72	2.22	6.33	5.81	6.90	2.60	2.32	2.91	5.86	2.08	16.52

CI = confidence intervals, RR = relative ratio. ^∗^All covariates were adjusted.

## 4 Discussion

To the best of our knowledge, this study is the first report examining trends and age-related characteristics of substance use (opioid, cocaine, marijuana, and heroin) hospitalizations among homeless individuals compared to all other patients. The main findings are monotonically increasing utilization patterns of substance use associated hospitalizations except cocaine among both homeless patients and all other patients, hospitalized homeless patients have high risks of substance use with 23% higher in opioid, 155% higher in cocaine, 43% higher in marijuana, and 57% higher in heroin than all other hospitalized patients, which is consistent with some previous studies.^[3,9]^ They may be explained by several underlying factors. First, homeless population has a higher probability of substance use than the general population.^[4,22]^ Vulnerable underlying health conditions among homeless individuals trig them to be hospitalized due to substance use compared to the general population.^[17]^ For example, “accelerated aging process” in older homeless individuals remain at risk of major cardiovascular events resulting from cocaine use and subsequent geriatric conditions and nursing home placement compared to the general population.^[17]^ Second, an unsanitary substance use environment may drive adverse effects. Previous studies reported that unsheltered homeless individuals are prone to increase the likelihood of exposure to communicable diseases^[4]^ and to be sexually victimized, especially, women homeless individuals.^[15]^ Although heroin use hospitalization rates are relatively low among homeless patients, the rate of hepatitis C infection is under recognized among injection drug user by sharing injection needles.^[23,24]^ According to the Center for Disease Control and Prevention estimation, actual hepatitis C infection cases are estimated to be 13.9 times the reported cases.^[11]^ The incidence rate of HCV infection was reported to be a cumulative incidence of 28% at 1 year of continuous drug injection.^[25]^ Policies and programs aiming to enhance recognition of hepatitis C infection among homeless individuals are urgently warranted to secure public health safety. For example, default opt-out option for hepatitis C screening from experiences of the default organ donation policy can be applied to older homeless individuals at safety-net public hospitals and public owned/operated shelters according to the Center for Disease Control and Prevention guideline of hepatitis C screening for baby boomer cohort born 1945 to 1965.^[26]^ Third, unintended negative consequences of substance use such as loss of job, loss of social and benefit support, financial hardship, and risk of imprisonment lead to chronic homelessness in later life.^[27]^ Further, the relationship between homelessness and substance use is complex and bidirectional.^[16,28]^ Substance abuse poses homeless individuals hard to rehabilitate to the society.^[9,28,29]^

The Affordable Care Act Medicaid expansion has led to the improvement of health care access and coverage for homeless individuals, especially for those with mental health and addiction conditions, main contributors to substance use.^[30–32]^ Our findings suggest that mental health conditions exist among one third of homeless patients, which is virtually the same as being reported by an earlier study based on the HCUP 10 state SIDs data in 2008.^[33]^ The Veterans Affairs health care system's innovative and compassionate services such as the “Hoptel” permanent supportive housing program is highly successful in reducing chronic homelessness among veterans.^[34]^ The Washington State Department of Commerce Housing Strategic Plan program made 1 in 4 homeless individuals get into permanent housing in 2016. However, programs or policies for interstate homeless individuals and neighboring effects from out-of-state homeless individuals remain wilderness,^[35]^ which suggests that more cooperative interstate regional policy support targeting substance using homeless individuals is needed.

Our findings also point out vulnerable age subgroups particularly among those younger than 20 years old or 60 years old or older among the homeless individuals. These 2 groups have much higher risks of using substances compared to nonsubstance use patients in hospitals. It is well known that age affects the health behaviors of the homeless individuals.^[36]^ Chronic homelessness is related to higher consumption rate of substance use.^[28,37]^ Contemporary old homeless individuals, many of them being baby boomers, experienced the crack cocaine epidemic when they were young.^[19]^ Children and young adult homeless individuals are known to be more tempted into substance use. Previous studies have found that more than half of adolescent homeless persons experience substance abuse.^[38]^ Lack of parenting, illicit drug user peer, mental health conditions, subsequently high risks of expulsion from schools and imprisonment are main contributors to youth homeless’ substance abuse and chronic homelessness.^[39,40]^ Preventing homelessness programs targeting at youth and young adults in transitioning out-of-the criminal justice system or psychiatric facilities might be effective ways.

Our study has limitations. First, we used the SID of 3 states where different in demography, health policies, and health services markets to reduce overrepresentation from neighboring exist. Controlling for state might not be able to fully reflect those differences in the analysis. Second, given the data limitation, we were unable to distinguish the purposes of opioid and marijuana use as recreational, medical, or illicit. Third, the SID dataset might have nature of under-reporting of homeless individuals. For example, the annual average number of homeless individuals in state of Washington in SID was only 72. There was wide variation in estimating homeless individuals in state of Washington between 6904 from federal agency^[1]^ and 122,000 from state agency. Although the 2005 Homeless Housing and Assistance, RCW 43.185C in state of Washington gradually achieved to reduce number of homeless individuals, the annual rate of homeless among hospitalized patients in Washington was about 1.04% that was very low compared to 31.82% in Florida. Fourth, we could not specify the types of homeless status; first, transient, chronic, interstate, or former. This limitation may hinder our findings to offer more specific information to assist allocation of limited resources to public health investment and policy interventions.

In conclusion, homeless status poses higher risks of substance use hospitalized patients than that of other hospitalized patients across all substance types, opioid, cocaine, marijuana, and heroin. The continuous trends of increase in substance use in all type of substances except cocaine are observed regardless of homeless status among hospitalized patients. Old homeless patients are more vulnerable to hard drugs including cocaine and heroin, compared to their younger counterparts.

## Author contributions

**Conceptualization:** Sung Youn Chun, Ji Yoo, Jinwook Hwang, Pearl C. Kim, Jay J. Shen.

**Data curation:** Hyeki Park, Jay J. Shen.

**Formal analysis:** Sung Youn Chun, Hyeki Park.

**Methodology:** Sung Youn Chun, Hyeki Park, Jinwook Hwang, Jay J. Shen.

**Project administration:** Jay J. Shen.

**Supervision:** Jay J. Shen.

**Writing – original draft:** Sung Youn Chun, Hyeki Park, Ji Yoo, Jinwook Hwang, Pearl C. Kim, Seong Park, Jay J. Shen.

**Writing – review & editing:** Sung Youn Chun, Hyeki Park, Ji Yoo, Jinwook Hwang, Seong Park, Jay J. Shen.

## Supplementary Material

Supplemental Digital Content

## Supplementary Material

Supplemental Digital Content

## Supplementary Material

Supplemental Digital Content

## Supplementary Material

Supplemental Digital Content

## Supplementary Material

Supplemental Digital Content

## Supplementary Material

Supplemental Digital Content
