# Supplementary material for: Trends and age-related characteristics of substance use in the hospitalized homeless population
Source: Medicine (Baltimore). 2022 Feb 25;101(8):e28917. doi: 10.1097/MD.0000000000028917 (PMC8878700; doi:10.1097/MD.0000000000028917)
Supplement: Supplemental Digital Content [file medi-101-e28917-s001.docx]

Supplemental digital content 1. ICD-9-CM codes of substance use and mental health conditions

|  | Descriptions | ICD-9-CM codes |
| --- | --- | --- |
| Substance use (abuse, dependence, unspecified, poisoning) | Opioid | 304.00, 304.01, 304.02, 304.70, 304.71, 304.72, 305.50, 305.51, 305.52, 965.00, 965.02, 965.09, E850.1, E850.2, E935.1, E935.2 |
|  | Cocaine | 304.20, 304.22, 305.60, 305.61, 305.62, 970.81, 970.89 |
|  | Marijuana | 304.30, 304.31, 305.20, 305.21, 305.22 |
|  | Heroin | 965.01, E850.0, E935.0 |
| Mental health conditions | Mood disorders | 296.00, 296.01, 296.02, 296.03, 296.04, 296.05, 296.06, 296.10, 296.11, 296.12, 296.13, 296.14, 296.15, 296.16, 296.20, 296.21, 296.22, 296.23, 296.24, 296.25, 296.26, 296.30, 296.31, 296.32, 296.33, 296.34, 296.35, 296.40, 296.41, 296.42, 296.43, 296.44, 296.45, 296.46, 296.50, 296.51, 296.53, 296.54, 296.55, 296.56, 296.60, 296.61, 296.62, 296.63, 296.64, 296.65, 296.66, 296.7, 296.80, 296.81, 296.82, 296.89, 296.90, 296.99 |
|  | Schizophrenia, other non-mood psychotic disorders | 297.0, 297.1, 297.2, 297.3, 297.8, 297.9, 298.0, 298.1, 298.2, 298.3, 298.4, 298.8 |
|  | Anxiety, stress-related, somatoform disorders | 300.00, 300.01, 300.02, 300.09, 300.10, 300.11, 300.12, 300.13, 300.14, 300.15, 300.16, 300.19, 300.20, 300.21, 300.22, 300.23, 300.29, 300.3, 300.4, 300.5, 300.6, 300.7, 300.81, 300.82, 300.89, 301.51, 308.0, 308.1, 308.2, 308.3, 308.4, 309.1, 309.21, 309.24, 309.3, 309.8, 309.81, 309.82, 309.83, 309.89, 309.9 |
|  | Personality and factitious disorders | 301.0, 301.11, 301.12, 301.13, 301.20, 301.21, 301.22, 301.3, 301.4, 301.50, 301.51, 301.59, 301.6, 301.7, 301.81, 301.82, 301.83, 301.84, 301.89. 312.30, 312.39 |

ICD-9-CM; International Classification of Diseases, 9^th^ revision, Clinical Modification.
