# Supplementary material for: Trends and age-related characteristics of substance use in the hospitalized homeless population
Source: Medicine (Baltimore). 2022 Feb 25;101(8):e28917. doi: 10.1097/MD.0000000000028917 (PMC8878700; doi:10.1097/MD.0000000000028917)
Supplement: Supplemental Digital Content [file medi-101-e28917-s002.docx]

| Supplemental digital content 2. Characteristics comparisons of homeless and general population | | | | | | | |
| --- | --- | --- | --- | --- | --- | --- | --- |
|  |  |  | Homeless status | | | General population | |
|  |  | Total (N) | | N | % | N | % |
| **Sex** |  |  | |  |  |  |  |
|  | Male | 14031405 | | 76627 | 78.34 | 13954778 | 43.52 |
|  | Female | 18131534 | | 21192 | 21.66 | 18110342 | 56.48 |
| **Age (year-old)** | | | | | | | |
|  | Less than 20 | 4961260 | | 1528 | 1.56 | 4959732 | 15.47 |
|  | 20-39 | 5959044 | | 25963 | 26.54 | 5933081 | 18.50 |
|  | 40-59 | 7059657 | | 57279 | 58.56 | 7002378 | 21.84 |
|  | 60 or older | 14182978 | | 13049 | 13.34 | 14169929 | 44.19 |
| **Ethnicity/Race** | | | | | | | |
|  | White | 21371183 | | 63016 | 64.42 | 21308167 | 66.45 |
|  | Black | 4271177 | | 19330 | 19.76 | 4251847 | 13.26 |
|  | Hispanic | 5104948 | | 12481 | 12.76 | 5092467 | 15.88 |
|  | Asian and other | 1415631 | | 2992 | 3.06 | 1412639 | 4.41 |
| **Mental health conditions** | | | | | | | |
|  | No | 28169801 | | 64633 | 66.07 | 28105168 | 87.65 |
|  | Yes | 3993138 | | 33186 | 33.93 | 3959952 | 12.35 |
| **Number of comorbidities** | | | | | | | |
|  | High (4 or more) | 7438464 | | 19641 | 20.08 | 7418823 | 23.14 |
|  | Medium (2 or 3) | 9905617 | | 38785 | 39.65 | 9866832 | 30.77 |
|  | Low (0 or 1) | 14818858 | | 39393 | 40.27 | 14779465 | 46.09 |
| **Marijuana legalization** | |  | |  |  |  |  |
|  | After | 12391230 | | 33887 | 34.64 | 12357343 | 38.54 |
|  | Before | 19771709 | | 63932 | 65.36 | 19707777 | 61.46 |
| **State** | | | | | | | |
|  | Arizona | 5916153 | | 8303 | 8.49 | 5907850 | 18.42 |
|  | Florida | 23019199 | | 88868 | 90.85 | 22930331 | 71.51 |
|  | Washington | 3227587 | | 648 | 0.66 | 3226939 | 10.06 |
| **Year** | | | | | | | |
|  | 2007 | 2542170 | | 7686 | 7.86 | 2534484 | 7.90 |
|  | 2008 | 3324471 | | 8544 | 8.73 | 3315927 | 10.34 |
|  | 2009 | 3365482 | | 8876 | 9.07 | 3356606 | 10.47 |
|  | 2010 | 3929124 | | 8972 | 9.17 | 3920152 | 12.23 |
|  | 2011 | 3975987 | | 10549 | 10.78 | 3965438 | 12.37 |
|  | 2012 | 3975238 | | 11049 | 11.30 | 3964189 | 12.36 |
|  | 2013 | 3949345 | | 13062 | 13.35 | 3936283 | 12.28 |
|  | 2014 | 4029341 | | 15718 | 16.07 | 4013623 | 12.52 |
|  | 2015 | 3071781 | | 13363 | 13.66 | 3058418 | 9.54 |
| **Total** | | 32162939 | | 97819 | 100 | 32065120 | 100 |
