# Supplementary material for: Trends and age-related characteristics of substance use in the hospitalized homeless population
Source: Medicine (Baltimore). 2022 Feb 25;101(8):e28917. doi: 10.1097/MD.0000000000028917 (PMC8878700; doi:10.1097/MD.0000000000028917)
Supplement: Supplemental Digital Content [file medi-101-e28917-s006.docx]

Supplemental digital content 6. Annual rate of heroin-related hospitalizations per 100,000 hospitalizations
